# Emotional and instrumental social support and older adults’ depressive symptoms: collaborative individual participant data meta-analysis of 11 population-based studies of aging

**DOI:** 10.1093/aje/kwaf137

**Published:** 2025-07-10

**Authors:** Suraj Samtani, Gowsaly Mahalingam, Ben C P Lam, Darren M Lipnicki, Katya Numbers, Maria Fernanda Lima-Costa, Sergio Luis Blay, Erico Castro Costa, Shifu Xiao, Steffi Reidel-Heller, Susanne Röhr, Alexander Pabst, Nikolaos Scarmeas, Mary Yannakoulia, Mary Kosmidis, Murali Krishna, Kalyanaraman Kumaran, Suzana Shahar, Tze Pin Ng, Roger Ho, Ki-Woong Kim, Ingmar Skoog, Jenna Najar, Therese Rydberg Sterner, Mary Ganguli, Chung-Chou Ho Chang, Tiffany F Hughes, Perminder S Sachdev, Henry Brodaty, For the Cohort Studies of Memory in an International Consortium (COSMIC)

**Affiliations:** Centre for Healthy Brain Ageing (CHeBA), Discipline of Psychiatry and Mental Health, School of Clinical Medicine, UNSW Sydney, NSW 2052, Australia; Centre for Healthy Brain Ageing (CHeBA), Discipline of Psychiatry and Mental Health, School of Clinical Medicine, UNSW Sydney, NSW 2052, Australia; Centre for Healthy Brain Ageing (CHeBA), Discipline of Psychiatry and Mental Health, School of Clinical Medicine, UNSW Sydney, NSW 2052, Australia; School of Psychology and Public Health, La Trobe University, Melbourne, Australia; Centre for Healthy Brain Ageing (CHeBA), Discipline of Psychiatry and Mental Health, School of Clinical Medicine, UNSW Sydney, NSW 2052, Australia; Centre for Healthy Brain Ageing (CHeBA), Discipline of Psychiatry and Mental Health, School of Clinical Medicine, UNSW Sydney, NSW 2052, Australia; René Rachou Research Center, Oswaldo Cruz Foundation, Belo Horizonte, Brazil; Department of Psychiatry, Federal University of Sao Paulo—UNIFESP, Sao Paulo, Brazil; Center for Studies in Public Health and Aging, René Rachou Research Center, Belo Horizonte, Brazil; Department of Geriatric Psychiatry, Shanghai Mental Health Center, Shanghai Jiaotong University School of Medicine, 600 South Wan Ping Road, Shanghai 200030, China; Faculty of Medicine, Institute of Social Medicine, Occupational Health and Public Health (ISAP), University of Leipzig, Leipzig, Germany; Centre for Healthy Brain Ageing (CHeBA), Discipline of Psychiatry and Mental Health, School of Clinical Medicine, UNSW Sydney, NSW 2052, Australia; Faculty of Medicine, Institute of Social Medicine, Occupational Health and Public Health (ISAP), University of Leipzig, Leipzig, Germany; Global Brain Health Institute (GBHI), Trinity College Dublin, Dublin, Ireland; School of Psychology, Massey University, Albany Campus, Auckland, New Zealand; Faculty of Medicine, Institute of Social Medicine, Occupational Health and Public Health (ISAP), University of Leipzig, Leipzig, Germany; 1st Department of Neurology, Aiginition Hospital, National and Kapodistrian University of Athens, 72 Vasilisis Sofias Avenue, Athens 11528, Greece; Department of Neurology, The Gertrude H. Sergievsky Center, Taub Institute for Research in Alzheimer’s Disease and the Aging Brain, Columbia University, New York, NY, United States; Department of Nutrition and Dietetics, School of Health Sciences and Education, Harokopio University, Athens, Greece; Laboratory of Neuropsychology & Behavioral Neuroscience, School of Psychology, Aristotle University of Thessaloniki, Thessaloniki 54124, Greece; Institute of Public Health, Bangalore 560070, India; The Lifecourse Epidemiology Unit, University of Southampton, Southampton, United Kingdom; Faculty of Health Sciences, Community Rehabilitation and Aging Research Centre, Universiti Kebangsaan Malaysia, Jalan Raja Muda Abdul Aziz, 43000 Kuala Lumpur, Malaysia; Department of Psychological Medicine, Yong Loo Lin School of Medicine, National University of Singapore, Level 9, NUHS Tower Block, 1E Kent Ridge Road, 119228, Singapore; Department of Psychological Medicine, Yong Loo Lin School of Medicine, National University of Singapore, Level 9, NUHS Tower Block, 1E Kent Ridge Road, 119228, Singapore; Department of Neuropsychiatry, Seoul National University Bundang Hospital, 300 Gumiro, Bundanggu, Seongnamsi, Gyeonggido 463-707, Seoul, South Korea; Department of Psychiatry, Seoul National University College of Medicine, Seoul, South Korea; Department of Brain and Cognitive Science, Seoul National University College of Natural Sciences, Seoul, South Korea; Department of Psychiatry and Neurochemistry, Neuropsychiatric Epidemiology Unit, Institute of Neuroscience and Physiology, The Sahlgrenska Academy, Centre for Ageing and Health (AGECAP), University of Gothenburg, Mölndal, Sweden; Region Västra Götaland, Sahlgrenska University Hospital, Psychiatry, Cognition and Old Age Psychiatry Clinic, Gothenburg, Sweden; Department of Psychiatry and Neurochemistry, Neuropsychiatric Epidemiology Unit, Institute of Neuroscience and Physiology, The Sahlgrenska Academy, Centre for Ageing and Health (AGECAP), University of Gothenburg, Mölndal, Sweden; Region Västra Götaland, Sahlgrenska University Hospital, Psychiatry, Cognition and Old Age Psychiatry Clinic, Gothenburg, Sweden; Department of Psychiatry and Neurochemistry, Neuropsychiatric Epidemiology Unit, Institute of Neuroscience and Physiology, The Sahlgrenska Academy, Centre for Ageing and Health (AGECAP), University of Gothenburg, Mölndal, Sweden; Department of Psychiatry, Epidemiology and Neurology, University of Pittsburgh, Pittsburgh, PA, United States; Department of Medicine, School of Medicine, University of Pittsburgh, Pittsburgh, PA, United States; Youngstown State University, Youngstown, OH, United States; Centre for Healthy Brain Ageing (CHeBA), Discipline of Psychiatry and Mental Health, School of Clinical Medicine, UNSW Sydney, NSW 2052, Australia; Centre for Healthy Brain Ageing (CHeBA), Discipline of Psychiatry and Mental Health, School of Clinical Medicine, UNSW Sydney, NSW 2052, Australia

**Keywords:** depression, late life, older adults, social support, protective

## Abstract

Social support is considered a protective factor against depression, but there are inconsistent findings regarding social support and depression in older adults. We aimed to clarify the association between emotional and instrumental social support and depressive symptoms in older adults cross-sectionally and longitudinally (mean follow-up = 1.96 years). We meta-analyzed raw individual participant level data from adults in mid- and late life (*N* = 23 973) who completed questionnaires about physical health, mental health, and social support and completed neuropsychological assessments. These were COSMIC (Cohort Studies of Memory in an International Consortium) cohort studies carried out in Australia, Brazil, China, Germany, Greece, India, Indonesia, Singapore, South Korea, Sweden, and the United States in mostly urban settings. After controlling for depression risk factors, emotional support (*B* = −0.40 [95% CI, −0.60 to −0.21]), but not instrumental support (*B* = 0.17 [95% CI, −0.26 to 0.59]), was associated with lower depressive symptoms cross-sectionally and at follow-up [emotional support (*B* = −0.37 [95% CI, −0.54 to −0.20]); instrumental support (*B* = 0.09 [95% CI, −0.30 to 0.49])]. Emotional support was associated with lower depressive scores cross-sectionally and longitudinally, while instrumental support was not associated with depressive symptoms. Our findings can help inform the nature of interventions to prevent and reduce risk of depression among older adults.

**This article is part of a Special Collection on Cross-National Gerontology**.

## Introduction

Depression affects over 25 million people worldwide and its global burden has increased almost 50% since 1990-2017.^1^ Late-life depression is associated with increased risk of cardiovascular diseases, cancer, autoimmune diseases, and dementia.^2^ Several environmental, biological, psychosocial, and behavioral risk factors are implicated in the etiology of depression.^3^ The stress-diathesis model, one of the leading conceptual models of depression,^4^ suggests that genetic predisposition combined with life stressors manifests as depressive symptoms.

Social support may have an important role in managing stressors and reducing the risk of depression. Social support promotes well-being and resilience.^5^^,^^6^ A recent systematic review of cohort studies identified that general social support from spouses, family, and friends is protective against depression among adults and older adults cross-sectionally and longitudinally.^7^

All types of social support may not be equally helpful. Social support could be instrumental (practical help with daily tasks) or emotional (having someone to confide in when stressed) in nature. There is evidence to support the hypothesis that emotional support is associated with lower levels of depressive symptoms in older adults, but the evidence linking instrumental support and lower levels of depressive symptoms is inconsistent. A systematic review focusing on older adults from Western countries found that instrumental support was *not* associated with lower levels of depressive symptoms, but emotional support was in 1 of the 2 studies.^7^ High perceived emotional social support from family, significant others, and friends was associated with significantly lower depression levels among American older adults.^8^ Notably, levels of support were higher for those who lived with others. In another study, high emotional social support was found to be associated with lower levels of depressive symptoms among American older adults.^9^ Among Chinese older adults, emotional (*r* = −0.31, *P* <.01), but not instrumental (*r* = 0.09) support from children was associated with lower levels of depressive symptoms.^10^ The association between social support and depressive symptoms is underinvestigated among older adults from non-Western countries. Social support, including emotional and instrumental support, were associated with lower levels of depressive symptoms among community-dwelling older adults in Asia.^11^ In summary, the association between instrumental support and lower levels of depressive symptoms has not been found in Western countries, and has only been found in 1 study with an Asian sample. Cultural differences may exist but need further exploration. Thus, we sought to explore the association between social support and depressive symptoms across multiple cohorts from around the globe.

Notably, there is a paucity of meta-analyses on the association between social support and depressive symptoms among older adults. One systematic review and meta-analysis of 100 eligible studies from Western countries found that older adults relied more on spouses, and that support from friends and having good spousal support were protective against depression.^7^ The study, however, relied on aggregate data from studies controlling for differing sets of variables, with mostly cross-sectional studies from exclusively Western countries.

Some studies have also examined sex differences in the association between social support and depressive symptoms. Women may tend to get support from a wider network while men tend to rely almost exclusively on spouses.^12^ A longitudinal study of Korean older adults revealed, however, that a lack of emotional support was associated with more depressive symptoms for both men and women.^13^ Thus, there is limited exploration of sex differences in the association between social support and depressive symptoms and no evidence of sex differences in this association to date.

In the current study, we examined the associations between social support (emotional and instrumental) and depressive symptoms cross-sectionally and longitudinally. This study involved a collaboration between 11 longitudinal cohort studies of aging from the Cohort Studies of Memory in an International Consortium (COSMIC^14^). Unlike a traditional systematic review and pooled meta-analysis, an individual participant data meta-analysis does not involve a systematic review as it accesses raw data. It allows us to control for the same variables and harmonize predictors, covariates, and outcomes across studies to allow firmer cross-nation comparisons. Furthermore, we accessed data from longitudinal studies from around the world.

We hypothesized that emotional support, but not instrumental support, would be related to lower levels of depressive symptoms both cross-sectionally and longitudinally across the cohort studies.

## Methods

### Participants

Individual participant data (IPD) were provided by the population-based longitudinal studies of aging shown in Table 1; all are members of COSMIC.^14^

**Table 1:** Sample characteristics for each of the 11 cohort studies.

**Study name**	**Acronym**	**Country**	**Sample size at baseline**	**Mean age (Range)**	**Sex (% Female)**	**Years of education Mean (SD)**	**Baseline & follow-up year**
Bambui cohort study of aging^15^	BAMBUI	Brazil	1510	68.98 (60.00-95.00)	61.06	2.75 (2.97)	1997, 1999
Chinese longitudinal study of aging^16^	CLAS	China	3127	71.55 (55.00-97.00)	54.86	8.18 (5.36)	2011, 2012
Gothenburg H70 birth cohort studies^17^	H70	Sweden	1015	75.26 (70.02-93.89)	77.44	9.66 (4.09)	2000-2004, 2005
Hellenic longitudinal investigation of aging & diet^18^	HELIAD	Greece	2020	73.09 (54.00-93.00)	59.69	8.02 (5.02)	2011, 2014
Korean longitudinal study on cognitive aging and dementia^19^	KLOSCAD	South Korea	6414	70.13 (58.00-101.00)	56.65	8.42 (5.34)	2009-2012, 2012-2014
Leipzig longitudinal study of the aged^20^	LEILA75+	Germany	1076	81.76 (75.11-99.29)	75.46	11.96 (1.78)	1996-1998,1998-1999
Neuroprotective model for healthy longevity among Malaysian older adults towards using aging^21^	LRGS TUA	Malaysia	1472	68.77 (60.00-91.00)	50.82	5.11 (3.91)	2013-2014, 2016-2017
Sydney memory and aging study^22^	MAS	Australia	1032	78.85 (70.30- 90.80)	55.04	11.60 (3.48)	2005-2007, 2007-2009
Monongahela-Youghiogheny healthy aging team^23^	MYHAT	USA	1914	78.85 (70.30-99.00)	60.97	12.85 (2.42)	2006-2008, 2010 (follow-up 2)
Mysore studies of natal effects on aging, and health^24^	MYNAH	India	721	62.36 (55.00-80.51)	43.41	10.87 (4.11)	2017, N/A
Singapore longitudinal study of aging^25^	SLAS	Singapore	2795	66.02 (55.00-97.60)	63.15	6.64 (4.60)	2003-2004, 2005-2007
Overall	N/A	N/A	23 748	71.55 (54.00-101.00)	59.10	8.29 (5.28)	N/A

Abbreviation: N/A, not applicable.

### Measures

Measures of social support and depression, which differed across cohort studies, required harmonization (for abbreviations and data see Table 1 and Tables S1-S5). Both emotional and instrumental support were harmonized into binary variables. Instrumental support data were harmonized from social support items (if available) or items assessing Instrumental Activities of Daily Living (IADL): managing finances, transportation, shopping and meal preparation, housecleaning and home maintenance, managing communication, and/or managing medications. Instrumental support items were chosen if they indicated *received* support rather than only perceived support, for the sake of consistency across studies since most included received instrumental support. Out of the 11 cohorts that were included in this study, 7 included measures of emotional support and 9 included measures of instrumental support, while 5 included both support types.

Depression symptoms were assessed using rating scales such as the General Health Questionnaire (BAMBUI), Geriatric Depression Scale (CLAS, HELIAD, KLOSCAD, MAS, SLAS), Centre for Epidemiology Scale-Depression (MYHAT), or Euro-Depression scale (MYNAH). Depression scores were first log transformed to approximate normal distributions and then standardized against baseline within each cohort. We used the standardized scores (ie, *z* scores) as dependent variables in our analyses.

Global cognition was based on Mini-Mental State Examination (MMSE) scores, or Community Screening Instrument for Dementia scores converted to MMSE scores for MYNAH^26^ (see Table S6).

### Statistical analyses

We used multiple imputation to replace missing values for predictors and covariates with 20 imputations within each study. We employed the 2-stage IPD meta-analysis approach to pool the effects across studies.^27^^,^^28^ The first stage involved estimating the association between social support and depressive symptoms within each study (cross-sectionally and longitudinally).

The second stage involved computing an overall estimate across studies using random effects meta-analysis, with effects been pooled using Rubin’s rules. Heterogeneity was examined using the *I*^2^ statistic.^29^^,^^30^ Funnel plots were examined to explore the presence of bias across studies.^31^

We controlled for age and sex in partially adjusted models. In fully adjusted models, we also controlled for other risk factors for depression: years of education, living situation, smoking history, diabetes mellitus, cardiovascular risk, global cognitive function, and (where available) history of depression in both cross-sectional and longitudinal analyses (see Tables S7-S11).

The cross-sectional analyses were performed with baseline data using multiple regression, and the longitudinal analyses using linear mixed modeling with a random intercept for participant and random slope of time-in-study. The longitudinal analyses were based on a single follow-up wave for each study. We chose the follow-up closest to 2 years to minimize discrepancy across studies. As follow-up data on depression were not available in the MYNAH study, it was only included in the cross-sectional analyses.

We also ran sensitivity analyses with cohort studies that included both instrumental and emotional support to examine the effect of each type of social support while controlling for the other type of social support. This allowed us to understand if one type of social support was associated with lower depressive symptoms despite receiving the other type of social support.

Finally, we examined sex differences in the association between emotional/instrumental support and depressive symptoms. Specifically, we explored the interaction effect of emotional/instrumental support and sex while predicting depressive symptoms.

We used R software *mice* package for multiple imputation, *lme4* package for linear mixed models, and *metafor* for the meta-analyses.

All studies had institutional ethics approvals.

## Results

Table 1 presents the demographic characteristics of the participants. The overall mean age at baseline was 71.44 years (range = 54-99.41 years, SD = 8.12 years). Over half (58.82%) of the participants in each study were female (except MYNAH). The overall mean years of education was 8.34 years (range = 0-48 years, SD = 5.20 years). The overall mean follow-up time was 1.96 years (SD = 0.88 years).

Table 2 presents the descriptive statistics for social support and depressive symptoms. As shown in Table 2, most participants in the cohort studies reported having emotional support, whereas there was more variation in the proportion of participants receiving instrumental support. Notably 87% of KLOSCAD participants reported receiving instrumental support.

**Table 2:** Harmonized social support and depression variable statistics for each of the 11 cohort studies.

**Study**	**Country**	**Emotional support**		**Instrumental support**		**Depression (baseline) (SD)**	**Depression (follow-up) (SD)**
		**Yes (%)**	**No (%)**	**Yes (%)**	**No (%)**		
BAMBUI	Brazil			1396 (87)	208 (13)	0.02 (0.93)	0.05 (0.86)
CLAS	China	3044 (97)	99 (3)			0.02 (0.95)	−0.02 (0.86)
H70	Sweden	555 (64)	318 (36)			0.03 (0.94)	0.02 (0.95)
HELIAD	Greece	1416 (70)	604 (30)	113 (6)	1908 (94)	0.06 (0.85)	0.05 (0.87)
KLOSCAD	South Korea	5164 (81)	1248 (19)	5588 (87)	824 (13)	0.00 (0.99)	0.00 (0.99)
LEILA75+	Germany			477 (39)	744 (61)	0.00 (0.99)	0.00 (0.99)
LRGSTUA	Malaysia			1018 (45)	1246 (55)	0.01 (0.96)	0.01 (0.96)
MAS	Australia	928 (95)	51 (5)	70 (7)	931 (93)	0.02 (0.94)	0.02 (0.95)
MYHAT	USA	1864 (97)	51 (3)	680 (36)	1235 (64)	0.08 (0.77)	0.08 (0.70)
MYNAH	India			12 (2)	709 (98)	0.06 (0.83)	
SLAS	Singapore	2639 (95)	142 (5)	640 (23)	2151 (77)	0.05 (0.88)	0.06 (0.82)
Overall	N/A	15 610 (86)	2513 (14)	9956 (50)	9994 (50)	0.03 (0.93)	0.02 (0.91)

Abbreviation: N/A, not applicable.

### Separate models for emotional and instrumental support

**Figure 1:**
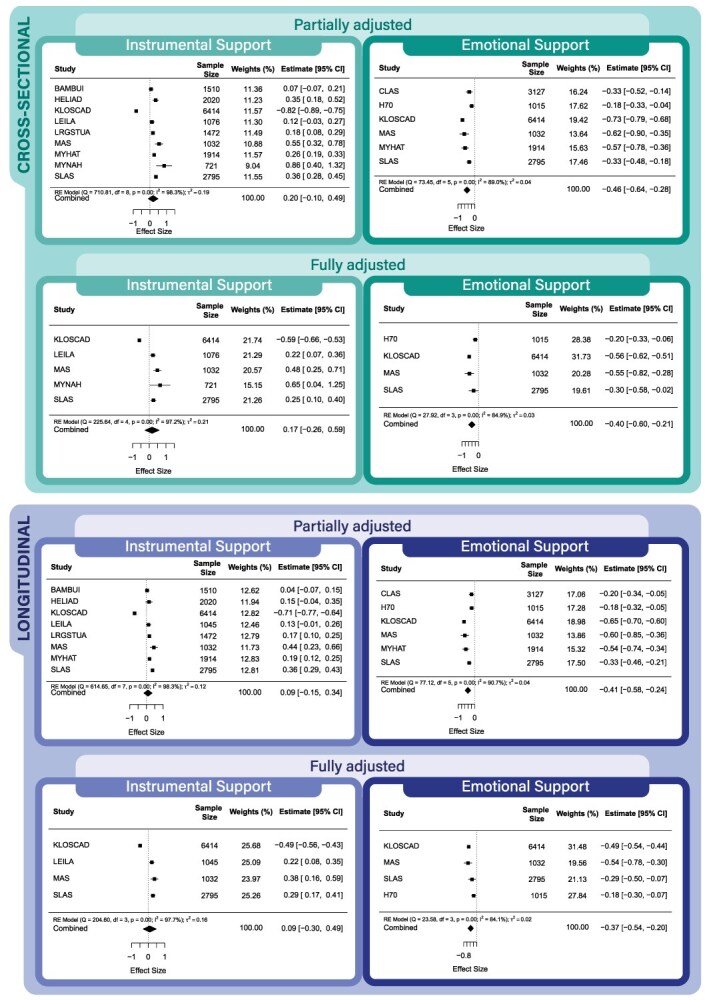
Partially and fully adjusted cross-sectional and longitudinal models for emotional or instrumental support and depressive symptoms.

Figure 1 presents results from the partially and fully adjusted cross-sectional and longitudinal models. Only emotional support was associated with having lower levels of depressive symptoms in these models. Funnel plots showed evidence of publication bias for some instrumental support models but not for any emotional support models (see Figure S1).

Heterogeneity was high (*I*^2^ range for fully adjusted models: 84.10%-97.74%), indicating significant variance between studies in the associations between social support and depressive symptoms. In the cross-sectional models, emotional support was associated with lower levels of depressive symptoms in all cohorts, whereas instrumental support was associated with lower levels of depressive symptoms in 1 cohort (South Korea) and with *more* depressive symptoms in other cohorts (Germany, Australia, and India). The pattern was repeated in the longitudinal models. Emotional support was again associated with lower levels of depressive symptoms in all cohorts, whereas instrumental support was associated with lower levels of depressive symptoms in 1 cohort (South Korea) and with *more* depressive symptoms in other cohorts (Germany and Australia but not India).

#### Sensitivity analysis for emotional support

The CLAS social support item (“In the past, when you encounter difficulties, what is the source that you ever received comfort and caring?”) may measure a mix of emotional and instrumental support. Therefore, to investigate the association between emotional support and depressive symptoms, we reran the emotional support partially adjusted models without the CLAS cohort. Our findings remained unchanged: Emotional support, cross-sectional, partially adjusted: −0.49 (95% CI, −0.70 to −0.28); Emotional support, longitudinal, partially adjusted: −0.46 (95% CI, −0.64 to −0.28).

#### Sensitivity analyses for instrumental support

We ran sensitivity analyses with cohorts that included direct questions about instrumental support (BAMBUI, KLOSCAD, HELIAD, MYHAT) rather than IADL items (LEILA75+, MAS, MYNAH, SLAS). This was only possible for the partially adjusted models, as the fully adjusted models only had data from KLOSCAD remaining. Our findings remained unchanged: Instrumental support, cross-sectional, partially adjusted: −0.01 [−0.41 to 0.43]; Instrumental support, longitudinal, partially adjusted: −0.03 [−0.37 to 0.31].

#### Sensitivity analyses excluding people with dementia at baseline

People with dementia at baseline may require higher levels of instrumental support. As such, we ran further sensitivity analyses excluding people with dementia at baseline (*N* = 1099 excluded; see Tables S12-S13). Our results remained unchanged for the cross-sectional and longitudinal models for emotional and instrumental support.

### Models with both emotional and instrumental support

**Figure 2:**
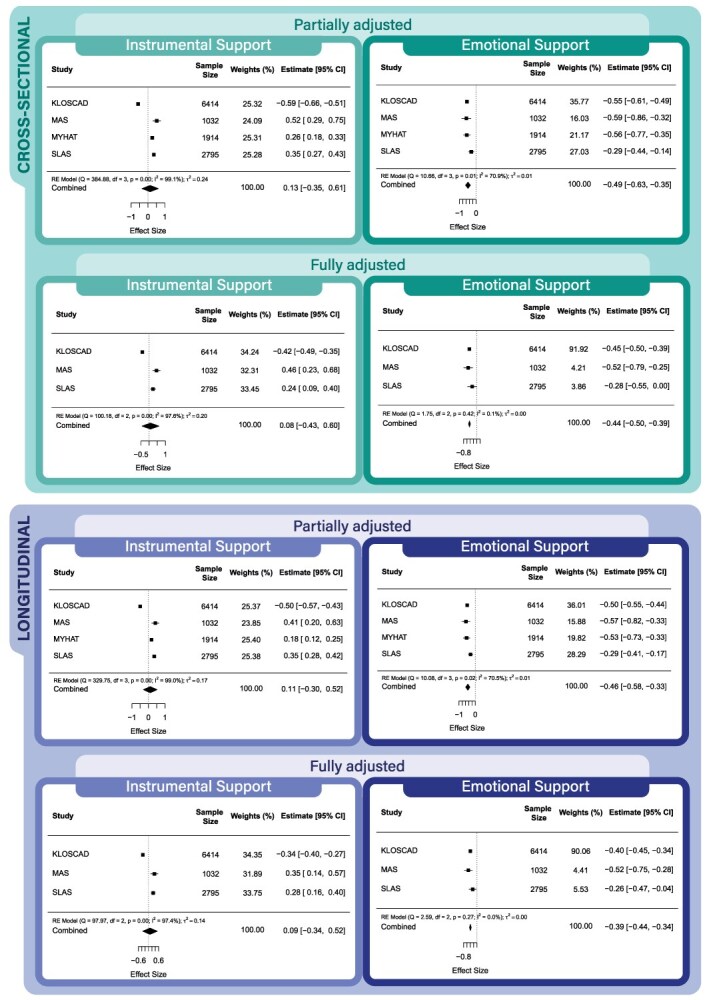
Partially and fully adjusted cross-sectional and longitudinal models for studies with both emotional and instrumental support and depressive symptoms.

Figure 2 presents partially and fully adjusted results from the cross-sectional and longitudinal models using data from studies with *both* emotional and instrumental support. While controlling for received instrumental support, emotional support was associated with lower levels of depressive symptoms cross-sectionally and longitudinally. However, receipt of instrumental support was not associated with lower levels of depressive symptoms while controlling for the presence of emotional support in any model. Funnel plots for these models are shown in Figure S2.

Heterogeneity indices varied greatly for the models with both types of social support (*I*^2^ range for fully adjusted models: 0%-97.56%), suggesting that the associations differed across cohorts. In the cross-sectional models, emotional support was associated with lower levels of depressive symptoms in all cohorts, whereas instrumental support was associated with lower levels of depressive symptoms in 1 cohort (South Korea) and *more* depressive symptoms in another cohort (Singapore). Similarly, in longitudinal models, emotional support was associated with lower levels of depressive symptoms in all cohorts, whereas instrumental support was associated with lower levels of depressive symptoms in 1 cohort (South Korea) and *more* depressive symptoms in 1 cohort (Australia).

#### Sensitivity analyses excluding people with dementia at baseline

As mentioned above, we ran sensitivity analyses excluding people with dementia at baseline (see Tables S12 and S13). The results for the cross-sectional and longitudinal remained unchanged.

### Sex differences

Table S14 shows the results for the sex differences in the association between social support indicators (either emotional or instrumental support) and depressive symptoms. There were no significant effects for the sex interaction term in any of the models, indicating that the association between emotional/instrumental support and depressive symptoms did not differ across males and females.

## Discussion

We investigated whether social support was associated with depressive symptoms in the second half of life using cross-sectional and longitudinal analyses of data from international cohort studies of aging. We found that emotional support, but not instrumental support, was associated with lower levels of depressive symptoms across all models: cross-sectional and longitudinal, partially and fully adjusted. This was also the case when we ran additional models with data only from cohort studies with data for both types of social support. This allowed us to evaluate the association between one type of social support and depressive symptoms, while controlling for the other type of social support.

The association between having emotional support and lower depressive symptoms could be explained by the buffering role of support against stress.^32^ Social support has been hypothesized to counter stress by inhibiting unhelpful coping behaviors and facilitating reappraisal of stressful situations and adaptive behaviors.^32^ A systematic review of mechanistic pathways by which social support protects against depression proposed that social relationships aid emotional processing of events.^33^ There is evidence that the lack of social support partially mediates the relationship between loneliness and depression.^34^ Biological mechanisms may also be responsible, as social isolation is hypothesized to interfere with the repair and maintenance of physiological functioning.^35^ Thus, a variety of cognitive (reappraisal), emotional (processing), and physiological (repair and maintenance) pathways may be responsible for the protective effects of social support against depression among older adults.

Instrumental support was associated with high levels of depressive symptoms in almost all cohorts. Individuals receiving instrumental support may be experiencing health conditions such as Mild Cognitive Impairment, dementia, arthritis, or diabetes mellitus. Receiving instrumental support may also be associated with feelings of helplessness and lack of autonomy. There may be more instrumental social support in some cultures or families in response to depressive symptoms.

Sex differences in the association between emotional or instrumental support and depressive symptoms were not statistically significant. This is in line with a previous longitudinal study that found that the association of lack of social support and more depressive symptoms was similar for men and women.^13^

The possibility of reverse causality cannot be ruled out. Social withdrawal is one of the key elements of depressive symptoms among older adults.^36^ Additionally, people who are depressed may have a reporting bias (ie, not see relationships as being supportive). Alternatively, both depressive symptoms and lack of social support may be linked to a third factor such as physical illness.

The binary nature of the harmonized social support variable available in most studies may have led to a loss of statistical power. We were unable to create multitiered harmonized variables, due to the presence of binary items in some studies. Future studies should include nonbinary questions to assess social support and its association with other aspects of health.

Our findings suggest that it may be important to promote emotional support among older adults in order to prevent or reduce depressive symptoms. This is in line with evidence that peer support interventions reduce depressive symptoms.^37^ Emotional support was associated with lower levels of depressive symptoms across all cohorts in all models. These associations were statistically significant in each cohort, despite heterogeneity in the strength of the associations between cohorts. The strongest associations were in the South Korean (95% CI, −0.70 to 0.60), Australian (95% CI, −0.85 to −0.36), and American cohorts (95% CI, −0.74 to −0.34), and slightly weaker in the Swedish (95% CI, −0.32 to −0.05) and Singaporean cohorts (−0.46 to −0.21). These results do not appear to cluster by world region; they may be explained by cultural differences within regions.

In contrast, instrumental support was only associated with lower levels of depressive symptoms in the South Korean cohort across models. The decline of intergenerational households in Asia^38^ may herald less frequent contact between adult children and their parents.^39^ However, instrumental support was not associated with lower levels of depressive symptoms in Singapore. We found high levels of instrumental support in the South Korean cohort (87%), but not in the Singaporean cohort (23%). It may be that cultural differences in the use and stigma of receiving instrumental support have influenced these results. It may be that receiving instrumental support is seen more as interdependence in South Korea and normalized, whereas it may be seen as “being a burden” in Singapore.

The current study has limitations. Heterogeneity in the questions measuring covariates, social support, and depression across cohorts limited the harmonization of emotional and instrumental support to binary variables, resulting in a loss of information. Small samples driving larger effect sizes and discrepancies in study design across cohorts are potential factors associated with this bias. Harmonization, however, allowed the inclusion of covariates and a comparison of the association between social support and depression across countries. We also chose to use a 2-year (or closest available) follow-up across studies, as social support levels in older adults have been shown to change substantially over a span of 3 years.^40^ A longer follow-up may be disconnected from baseline levels of social support, and longitudinal studies that track changes in social support over time are required to further investigate if changes in social support precede changes in mental health. It may be cumulative support over time that influences propensity toward depression, which we were unable to examine due to social support data being available only at baseline. It may be that social support has a proximal impact, with social support fluctuations being associated with changes in depressive symptoms. Future studies could examine the interplay between social variables (such as living alone and having social support) in predicting depressive symptoms among older adults.

In conclusion, we conducted meta-analyses of harmonized data from across 11 countries examining the association between each of emotional and instrumental social support and depressive symptoms among older adults. The availability of emotional support was associated with lower levels of depressive symptoms cross-sectionally and longitudinally. Public health policy requires a focus on identifying vulnerable older adults who lack social support via improved screening processes and primary prevention. This could entail fostering community social groups, intergenerational interactions, digital communities, and built environments that support greater community interaction. Promoting social support groups for depression among older adults could be a promising area for future interventions. There remains a need for more comprehensive longitudinal data on social support and mental health for older adults.

## Supplementary Material

Web_Material_kwaf137

## Data Availability

Data were provided by the contributing studies to COSMIC on the understanding and proviso that the relevant study leaders be contacted for further use of their data and additional formal data sharing agreements be made. Researchers can apply to use COSMIC data by completing a COSMIC Research Proposal Form available from https://cheba.unsw.edu.au/consortia/cosmic/research-proposals.
