# Supplementary material for: Emotional and instrumental social support and older adults’ depressive symptoms: collaborative individual participant data meta-analysis of 11 population-based studies of aging
Source: Am J Epidemiol. 2025 Jul 10;194(10):3041–9. doi: 10.1093/aje/kwaf137 (PMC12527255; doi:10.1093/aje/kwaf137)
Supplement: Web_Material_kwaf137 [file web_material_kwaf137.zip › AJE-00881-2024 supplementary info 20250611 updated.docx]

**Emotional and instrumental social support and older adults’ depressive symptoms: Collaborative individual participant data meta-analysis of 11 population-based studies of ageing**

**Samtani et al.**

**Supplementary Material**

Contents

[Table S1. STROBE statement checklist for cohort studies 2](#_Toc200543256)

[Table S2. Ethics approvals for each cohort study contributing to the current study’s dataset. 5](#_Toc200543257)

[Table S3. Emotional Support Harmonisation 7](#_Toc200543258)

[Table S4. Instrumental Support Harmonisation 8](#_Toc200543259)

[Table S5. Depression scores and depression history items from each cohort study. 10](#_Toc200543260)

[Table S6. Tests of global cognition at baseline 11](#_Toc200543261)

[Table S7. Education 12](#_Toc200543262)

[Table S8. Smoking Harmonisation 13](#_Toc200543263)

[Table S9 Cardiovascular Disease Harmonisation 14](#_Toc200543264)

[Table S10. Diabetes Mellitus Harmonisation 15](#_Toc200543265)

[Table S11. Living Situation Harmonisation 16](#_Toc200543266)

[Table S12. Identification of people with dementia 17](#_Toc200543267)

[Table S13. Sensitivity analyses excluding people with dementia at baseline 18](#_Toc200543268)

[Table S14. Sex differences models 20](#_Toc200543269)

[Figure S1. Funnel plot for the meta-analysis examining association between emotional and instrumental social support (separate models) and depression scores a) cross-sectionally and b) longitudinally. 21](#_Toc200543270)

[Figure S2. Funnel plot for the meta-analysis examining association between emotional and instrumental social support (combined model) and depression scores a) cross-sectionally and b) longitudinally. 22](#_Toc200543271)

[References for Supplementary Material 23](#_Toc200543272)

# Table S1. STROBE statement checklist for cohort studies

|  | **Item No** | **Recommendation** |
| --- | --- | --- |
| **Title and abstract** | 1 | (*a*) Indicate the study’s design with a commonly used term in the title or the abstract |
|  |  | Title |
|  |  | (*b*) Provide in the abstract an informative and balanced summary of what was done and what was found  Abstract |
| **Introduction** | | |
| Background/rationale | 2 | Explain the scientific background and rationale for the investigation being reported  Introduction: paragraphs 1-3 |
| Objectives | 3 | State specific objectives, including any prespecified hypotheses  Introduction, paragraph 4 |
| **Methods** | | |
| Study design | 4 | Present key elements of study design early in the paper  Methods |
| Setting | 5 | Describe the setting, locations, and relevant dates, including periods of recruitment, exposure, follow-up, and data collection  Methods  Table 1 |
| Participants | 6 | (*a*) Give the eligibility criteria, and the sources and methods of selection of participants. Describe methods of follow-up  Methods: Participants section  Table 1 |
|  |  | (*b*) For matched studies, give matching criteria and number of exposed and unexposed  NA |
| Variables | 7 | Clearly define all outcomes, exposures, predictors, potential confounders, and effect modifiers. Give diagnostic criteria, if applicable  Methods: Measures section.  S3-S11  Methods: Statistical Analyses section. |
| Data sources/ measurement | 8* | For each variable of interest, give sources of data and details of methods of assessment (measurement). Describe comparability of assessment methods if there is more than one group  Methods: Measures section.  Tables S3-S11 |
| Bias | 9 | Describe any efforts to address potential sources of bias  Methods: Statistical Analyses section  Figures S1-S2 |
| Study size | 10 | Explain how the study size was arrived at  Results: Sample section  Table 1 |
| Quantitative variables | 11 | Explain how quantitative variables were handled in the analyses. If applicable, describe which groupings were chosen and why  Methods: Statistical Analyses section  Tables S3-S11 |
| Statistical methods | 12 | (*a*) Describe all statistical methods, including those used to control for confounding  Methods: Statistical Analyses section. |
|  |  | (*b*) Describe any methods used to examine subgroups and interactions  Methods: Statistical Analyses section  Table S12 |
|  |  | (*c*) Explain how missing data were addressed  Methods: Statistical Analyses |
|  |  | (*d*) If applicable, explain how loss to follow-up was addressed  Methods: Statistical Analyses |
|  |  | (*e*) Describe any sensitivity analyses  Methods: Statistical Analyses |
| **Results** | | |
| Participants | 13* | *(a)* Report numbers of individuals at each stage of study—eg numbers potentially eligible, examined for eligibility, confirmed eligible, included in the study, completing follow-up, and analysed  Tables 1 |
|  |  | *(b)* Give reasons for non-participation at each stage  With 13 different contributing studies and varying numbers of assessment waves, this level of information is not appropriate here, though can be found via the references for the studies listed in Table 1. |
|  |  | *(c)* Consider use of a flow diagram  Thirteen different contributing studies and varying numbers of assessment waves preclude the use of a manageable flow diagram. |
| Descriptive data | 14* | *(a)* Give characteristics of study participants (eg demographic, clinical, social) and information on exposures and potential confounders  Tables 1-2 |
|  |  | *(b)* Indicate number of participants with missing data for each variable of interest  N/A |
|  |  | *(c)* Summarise follow-up time (eg, average and total amount)  Table 1 |
| Outcome data | 15* | Report numbers of outcome events or summary measures over time  Table 2 |
| Main results | 16 | *(a)* Give unadjusted estimates and, if applicable, confounder-adjusted estimates and their precision (eg, 95% confidence interval). Make clear which confounders were adjusted for and why they were included  Methods: Statistical Analyses section  Figures 1-3 |
|  |  | *(b)* Report category boundaries when continuous variables were categorized  Methods: Measures section.  Tables S3-S11 |
|  |  | *(c)* If relevant, consider translating estimates of relative risk into absolute risk for a meaningful time period  NA |
| Other analyses | 17 | Report other analyses done—eg analyses of subgroups and interactions, and sensitivity analyses  Sex differences models S12 |
| **Discussion** | | |
| Key results | 18 | Summarise key results with reference to study objectives  Discussion- paragraphs 1-4 |
| Limitations | 19 | Discuss limitations of the study, taking into account sources of potential bias or imprecision. Discuss both direction and magnitude of any potential bias  Discussion- paragraphs 5-6 |
| Interpretation | 20 | Give a cautious overall interpretation of results considering objectives, limitations, multiplicity of analyses, results from similar studies, and other relevant evidence  Discussion- paragraphs 1-3  Conclusion section |
| Generalisability | 21 | Discuss the generalisability (external validity) of the study results  Discussion: Strengths section |
| **Other information** | | |
| Funding | 22 | Give the source of funding and the role of the funders for the present study and, if applicable, for the original study on which the present article is based  Funding section |

# Table S2. Ethics approvals for each cohort study contributing to the current study’s dataset.

| **Study** | **Ethics approval** |
| --- | --- |
| Bambui Cohort Study of Ageing (BAMBUI) | Ethics Boards of the Fundac¸a˜o Oswaldo Cruz in Rio de Janeiro and the Instituto Rene´ Rachou of the Fundac¸a˜o Oswaldo Cruz in Belo Horizonte, Brazil (14/2007 - CEPSH-CpqRR) |
| Chinese Longitudinal Study of Ageing (CLAS) | Ethics Committee of Shanghai Mental Health Center (2011-YJ-14) |
| Gothenberg H70 Birth Cohort Studies (the H70 study) | Regional Ethical Review Board (No. 075-09) |
| Hellenic Longitudinal Investigation of Ageing & Diet (HELIAD) | Institutional Ethics Review Board of the University of Thessaly (ΒΕΥ846Ψ8Ν2-32Π) and of Aiginition Hospital (ΑΔΑ: ΒΕΥ846Ψ8Ν2-32Π) |
| Korean Longitudinal Study on Cognitive Aging and Dementia (KLOSCAD) | Institutional Review Board of Seoul National University Bundang Hospital, Korea (IRB No. B-0912/089-010) |
| Leipzig Longitudinal Study of the Aged (LEILA75+) | Ethics committee of the University of Leipzig (C7 79934700) |
| Neuroprotective Model for Healthy Longevity among Malaysian Older Adults Towards Using Ageing (LRGS TUA) | National University of Malaysia (UKM) research ethics committee (reference: UKM PPI/111/8/JEP-2019-024), and was also approved by the Malaysian National Medical Research and Ethics Committee at the Malaysian Ministry of Health [reference: KKM/NIHSEC/P19-1689(12)]. |
| Sydney Memory and Ageing Study (MAS) | University of New South Wales Human Research Ethics Committee (approval #14327) |
| Monongahela-Youghiogheny Healthy Aging Team (MYHAT) | University of Pittsburgh Human Research Protection Office (formerly Institutional Review Board, IRB). Approval # PRO16030244 |
| MYsore studies of Natal effects on Ageing and Health (MYNAH) | CSI Holdsworth Memorial Hospital Ethics Committee Board HMH/ERC/2012 |
| Singapore Longitudinal Study of Ageing (SLAS) | National University of Singapore Institutional Review Board (Reference Code: 04-140) |

Written consent was exclusively or predominantly obtained from participants in all studies. For LRGS TUA, verbal consent and thumbprint mark was obtained from illiterate people. Further participant consent was not deemed necessary as only fully de-identified data were shared with the analysis team (e.g., as per the Privacy Rule proposed by the National Institute of Health, USA: http://privacyruleandresearch.nih.gov/research_repositories.asp).

# Table S3. Emotional Support Harmonisation

| **Study** | **Coding: No = 0; Yes = 1** |
| --- | --- |
| BAMBUI | N/A |
| CLAS | Social Support Rating Scale Item 7: In the past, when you encounter difficulties, what is the source that you ever **received** comfort and caring?: Spouse; other family members; friends; relatives; colleagues; companies; official or semi-official organizations, such as, parties, leagues and trade union; unofficial organizations, such as, religion, social group and etc.; others. Lists at least one source = 1, no source = 0 |
| H70 | “Do you have more than one confidant to talk to?”: yes = 1, no = 0. |
| HELIAD | N/A |
| KLOSCAD | ‘Availability of someone to confide in’: none/usually none = 0, half/usually yes/yes = 1 |
| LEILA75+ | N/A |
| LRGSTUA | N/A |
| MAS | “Do you have someone in whom you can confide”: yes = 1, no = 0 |
| MYHAT | ‘Do you feel close enough to any family or friends that you could confide in them about any difficulties or concerns?’: yes = 1, no = 0 |
| MYNAH | N/A |
| SLAS | ‘Do you have someone to confide in?’: yes = 1, no = 0 |

# Table S4. Instrumental Support Harmonisation

| **Study** | **Coding: No = 0; Yes = 1** |
| --- | --- |
| BAMBUI | “What kind of help or assistance does your family offer you? (Family members who live or who do not live with the interviewee)” Money, house, clothes, food, company/personal care: lists at least one source = 1; no source = 0 |
| CLAS | N/A |
| H70 | N/A |
| HELIAD | Taking medication: has aid = 1; alone/unable = 0  Money use: has aid = 1; independent = 0 |
| KLOSCAD | ‘Perceived and objective support received from people’: none/usually none = 0, half/usually yes/yes = 1 |
| LEILA75+ | IADL items: medical use, perform household chores, use the telephone, take care of financial matters, use public transport, buy groceries, prepare meals: if any: impossible to do so independently = 1; if all: no difficulties/with difficulties = 0 |
| LRGSTUA | Various items (can you go you to buy daily necessities or clothes; can do housework; can manage money; goes to a distant place (over 100m); can prepare own food; can take own medicine): unable by themselves/able with assistance = 1; able by themselves = 0  Score of 1 on any item = 1; score of 0 on all items = 0 |
| MAS | AQoL-2 items: help with household tasks:  I can do none of these tasks by myself/ I cannot do most of these tasks unless I have help = 1;  I can do these tasks only very slowly and without help / I can do these tasks relatively easily without help / I can do all these tasks very quickly and efficiently without any help = 0. |
| MYHAT | “Does anyone regularly help you with things like household chores, shopping, repairs, etc?” yes, free help / yes, paid help = 1; no = 0 |
| MYNAH | Need for care: needs care much of the time/ needs care some of the time = 1; does not need care; they are able to do everything for themselves = 0  AND  Time spent assisting to communicate: more than 2 hours/ 1-2 hours / less than 1 hour = 1; no time = 0  OR  Time spent assisting to use transport: more than 2 hours/ 1-2 hours / less than 1 hour = 1; no time = 0  Score of 1 if Need for Care = 1 and either communication/transport assistance = 1;  Score of 0 if Need for care = 0 |
| SLAS | Multiple IADL questions (using telephone, travelling, shopping, preparing meals, housework, taking medicine, managing money): dependent / aided = 1; independent = 0  Score of 1 on any item = 1; score of 0 on all items = 0 |

# Table S5. Depression scores and depression history items from each cohort study.

| **Study** | **Depressive symptoms** | **History of Depression: 0 (no), 1 (yes)** |
| --- | --- | --- |
| BAMBUI | General health questionnaire 12 | N/A |
| CLAS | Geriatric Depression Scale (GDS) | N/A |
| The H70 study | MADRS score (Montgomery-Åsberg Depression Rating Scale) | History of depression (self-reported, minor or major depression) |
| HELIAD | Geriatric depression scale (GDS) | N/A |
| KLOSCAD | Korean Geriatric Depression Scale | N/A |
| LEILA75+ | CES-D score | History of Depression (self-reported) |
| LRGSTUA | GDS score | N/A |
| MAS | GDS score |  |
| MYHAT | Modified CES-D score | N/A |
| MYNAH | Euro-D total score | History of depression (self-reported) |
| SLAS | GDS-15 score | History of depression (self-reported) |

# Table S6. Tests of global cognition at baseline

| **Study** | **Global Cognition** |
| --- | --- |
| BAMBUI | MMSE Total |
| CLAS | MMSE Total |
| the H70 study | MMSE Total |
| HELIAD | MMSE Total |
| KLOSCAD | MMSE Total |
| LEILA75+ | MMSE Total |
| LRGSTUA | MMSE Total |
| MAS | MMSE Total |
| MYHAT | MMSE Total |
| MYNAH | CSI-D Total converted to MMSE scores (Crane et al. 2008) |
| SLAS | MMSE Total |

**Note:** MMSE = Mini-mental state examination. CERAD = Consortium to Establish a Registry for Alzheimer Disease. CSI-D = Cognitive State Interview- Dementia. SIDAM = Structured Interview for the diagnosis of Dementia of the Alzheimer type, multi-infarct dementia and dementias of other etiology according to ICD-10 and DSM-IV. TICSm = Telephone Interview for Cognitive Status modified. WAIS-R = Wechsler Adult Intelligence Scale- Revised. WAIS-III = Wechsler Adult Intelligence Scale- Third Edition. MMC = Mini-Mental Cabán. *Spikes removed from data before analysis. Trail Making Test timings were reversed so that higher values indicated better performance.

# Table S7. Education

| **Study** | **Years of Education** |
| --- | --- |
| BAMBUI | Years of schooling |
| CLAS | Years of education |
| H70 | Education years |
| HELIAD | Years of education |
| KLOSCAD | Years of formal education |
| LEILA 75+ | Years of education |
| LRGS TUA | Years of education |
| MAS | Years of education |
| MYHAT | Years of education |
| MYNAH | Education level of subject. Recoded categorical levels: Illiterate = 0; Primary = 4; Secondary = 10; College = 12; Diploma = 11; Graduate = 15; Postgraduate = 17 |
| SLAS | Years of education |

# Table S8. Smoking Harmonisation

| **Study** | **Current: 0 (no), 1 (yes)** |
| --- | --- |
| BAMBUI | Self-reported current smoker |
| CLAS | 1 or more cigarettes per day |
| The H70 study | Current cigarette smoker |
| HELIAD | Self-reported current smoking |
| KLOSCAD | Current smoking pack/day 1+ |
| LEILA75+ | 1 or more cigarettes per day |
| LRGSTUA | Whether or not participant currently smokes |
| MAS | In the last month, smoked tobacco regularly |
| MYHAT | Whether participant currently smokes |
| MYNAH | Self-reported current smoker |
| SLAS | Self-reported smoking at least once/month or more |

N/A: not available

# Table S9 Cardiovascular Disease Harmonisation

| **Study** | **Current: 0 (no), 1 (yes - meeting any is sufficient)** |
| --- | --- |
| BAMBUI | Self-reported history of angina or myocardial infarction |
| CLAS | Self-reported history of heart disease, angina or myocardial infarction |
| The H70 study | Self-reported myocardial infarction |
| HELIAD | Self-reported history of coronary disease, myocardial infarction, congestive heart failure, arrythmia any other heart disease |
| KLOSCAD | Self-reported history of angina or myocardial infarct |
| LEILA75+ | Self-reported heart failure or myocardial infarction |
| LRGSTUA | 1. Self-reported heart disease diagnosed by doctor, 2. Self-reported heart disease |
| MAS | Self-reported heart problems, heart attack, angina, atrial fibrillation or cardiac arrhythmia requiring treatment |
| MYHAT | Self-reported heart attack, congestive heart failure or cardiac arrest |
| MYNAH | Self-reported heart problem |
| SLAS | Presence of cardiac disease: ischemic heart disease, heart failure or atrial fibrillation |

# Table S10. Diabetes Mellitus Harmonisation

| **Study** | **Current: 0 (no), 1 (yes - meeting any is sufficient)** |
| --- | --- |
| BAMBUI | 1. Fasting blood glucose, 2. Treatment |
| CLAS | Self-reported history of diabetes |
| The H70 study | Self-reported history of diabetes |
| HELIAD | Self-reported history of diabetes |
| KLOSCAD | 1. Current status of diabetes, 2. Fasting glucose levels |
| LEILA75+ | Self-reported diabetes or treatment |
| LRGSTUA | 1. Self-reported diabetes, 2. Self-reported diabetes diagnosed by a doctor |
| MAS | 1. Fasting blood glucose, 2. Treatment for diabetes, 3. History of diabetes |
| MYHAT | Self-reported diabetes |
| MYNAH | Self-reported diabetes |
| SLAS | 1. Fasting blood glucose, 2. Treatment for diabetes, 3. History of diabetes |

Fasting blood glucose criteria are ≥126mg/dL or >7mmol/L. N/A: not available

# Table S11. Living Situation Harmonisation

| **Study** | **Lives alone = 0; Lives with at least one person = 1** |
| --- | --- |
| BAMBUI | Total number of people living in the household (includes all): 1 = 0, 2 or more = 1 |
| CLAS | ‘Over the past past, you …’ stay away from family and live alone = 0; often move the residence and most of time live together with strangers/live together with students, colleagues or friends/live together with family = 1 |
| The H70 study | Cohabitant: no or not cohabitant = 0, yes, cohabitant = 1 |
| HELIAD | Number of persons living with the participant: 0 = 0, 1 or more = 1  Number of household including yourself: 0 = 0 ; 1+ = 1 |
| KLOSCAD | Cohabitants: living alone = 0, with spouse/with other family members/with spouse and other family members = 1 |
| LEILA75+ | Presence of cohabitants - Baseline: lives alone = 0, lives with spouse or significant other/lives with other family members or lives with others but not family members = 1 |
| LRGSTUA | Living status: alone = 0; with others = 1 |
| MAS | Number of people lived with: 0 = 0, 1 or more = 1 |
| MYHAT | Number of cohabitants: 0 = 0, 2 or more = 1 |
| MYNAH | Living arrangements: Alone = 0; With spouse only / with adult children (+/- others) / other living arrangements = 1 |
| SLAS | Staying with others: alone = 0, others = 1 |

# Table S12. Identification of people with dementia

| **Study** | **Dementia diagnosis** | **Number of people with dementia at baseline** |
| --- | --- | --- |
| BAMBUI | MMSE < 14 | 67 |
| CLAS | Consensus diagnosis | 195 |
| The H70 study | Consensus diagnosis | 95 |
| HELIAD | Consensus diagnosis | 102 |
| KLOSCAD | Consensus diagnosis | 289 |
| LEILA75+ | Consensus diagnosis | 220 |
| LRGSTUA | MMSE ≤ 2 SD and IADL < 6 or Clinical Dementia Rating ≥ 1, if available | 25 |
| MAS | People with dementia at baseline were excluded, based on consensus diagnosis | 0 |
| MYHAT | Clinical Dementia Rating ≥ 1 | 23 |
| MYNAH | 10/66 studies Dementia diagnosis | 22 |
| SLAS | Consensus diagnosis | 61 |
| Overall | - | 1099 |

Note. In order to identify people with dementia, we used consensus diagnoses wherever available and used established cut-off scores for specific populations. Consensus diagnoses were available for most studies (except BAMBUI, MYHAT). For BAMBUI, published cut-off scores for probable cognitive impairment/dementia were used (the BAMBUI MMSE cut-off is 13/14^1^ For MYHAT, a Clinical Dementia Rating of 1 or greater was used to identify PLWD^2^. For MYNAH, the 10/66 dementia studies algorithm was used to identify people with dementia^3^.

# Table S13. Sensitivity analyses excluding people with dementia at baseline

| **Type of social support** | **Type of model** | **Sub-type of model** | **Effect** | **Heterogeneity** | **Tau** | **Eggers** |
| --- | --- | --- | --- | --- | --- | --- |
| Models with either instrumental or emotional support | **-** | **-** | **-** | **-** | **-** | **-** |
| Instrumental | Cross-sectional | Partially adjusted | 0.25 (-0.06, 0.57) | 98.11 | 0.22 | z = 2.17, p = 0.03 |
| Instrumental | Cross-sectional | Fully adjusted | 0.18 (-0.26, 0.62) | 97.11 | 0.23 | z = 1.10, p = 0.27 |
| Instrumental | Longitudinal | Partially adjusted | 0.09 (-0.15, 0.34) | 98.28 | 0.12 | z = 1.03, p = 0.30 |
| Instrumental | Longitudinal | Fully adjusted | 0.09 (-0.30, 0.49) | 97.74 | 0.16 | z = 2.01, p = 0.04 |
| Emotional | Cross-sectional | Partially adjusted | -0.45 (-0.63, -0.26) | 86.87 | 0.04 | z = 0.23, p = 0.82 |
| Emotional | Cross-sectional | Fully adjusted | -0.42 (-0.65, -0.19) | 87.24 | 0.04 | z = 0.35, p = 0.73 |
| Emotional | Longitudinal | Partially adjusted | -0.41 (-0.58, -0.24) | 90.71 | 0.04 | z = -0.10, p = 0.92 |
| Emotional | Longitudinal | Fully adjusted | -0.37 (-0.54, -0.20) | 84.10 | 0.02 | z = -0.01, p = 1.00 |
| Models with both instrumental and emotional support | - | - | - | - | - | - |
| Instrumental (Adjusting for emotional) | Cross-sectional | Partially adjusted | 0.12 (-0.36, 0.59) | 98.72 | 0.23 | z = 0.92, p = 0.36 |
| Emotional (Adjusting for Instrumental) | Cross-sectional | Partially adjusted | -0.45 (-0.63, -0.27) | 73.37 | 0.02 | z = 0.18, p = 0.85 |
| Instrumental (Adjusting for emotional) | Cross-sectional | Fully adjusted | 0.11 (-0.45, 0.67) | 97.35 | 0.24 | z = 4.70, p = 0.00 |
| Emotional (Adjusting for Instrumental) | Cross-sectional | Fully adjusted | -0.49 (-0.56, -0.42) | 0.06 | 0.00 | z = 0.77, p = 0.44 |
| Instrumental (Adjusting for emotional) | Longitudinal | Partially adjusted | 0.11 (-0.30, 0.52) | 99.02 | 0.17 | z = 0.72, p = 0.47 |
| Emotional (Adjusting for Instrumental) | Longitudinal | Partially adjusted | -0.46 (-0.58, -0.33) | 70.48 | 0.01 | z = -0.51, p = 0.61 |
| Instrumental (Adjusting for emotional) | Longitudinal | Fully adjusted | 0.09 (-0.34, 0.52) | 97.37 | 0.14 | z = 1.46, p = 0.14 |
| Emotional (Adjusting for Instrumental) | Longitudinal | Fully adjusted | -0.39 (-0.44, -0.34) | 0.00 | 0.00 | z = -0.03, p = 0.98 |

# Table S14. Sex differences models

| **Type of social support** | **Type of model** | **Sub-type of model** | **Effect size for interaction term (sex x social support)** |
| --- | --- | --- | --- |
| Models with either instrumental or emotional support | - | - | - |
| Instrumental | Cross-sectional | Partially adjusted | 0.02 (-0.05, 0.09) |
| Instrumental | Cross-sectional | Fully adjusted | -0.07 (-0.23, 0.08) |
| Instrumental | Longitudinal | Partially adjusted | 0.01 (-0.05, 0.07) |
| Instrumental | Longitudinal | Fully adjusted | -0.04 (-0.19, 0.11) |
| Emotional | Cross-sectional | Partially adjusted | 0.04 (-0.09, 0.16) |
| Emotional | Cross-sectional | Fully adjusted | 0.01 (-0.09, 0.11) |
| Emotional | Longitudinal | Partially adjusted | 0.05 (-0.05, 0.16) |
| Emotional | Longitudinal | Fully adjusted | 0.04 (-0.05, 0.12) |
| Models with both instrumental and emotional support | - | - | - |
| Instrumental (Adjusting for emotional) | Cross-sectional | Partially adjusted | -0.00 (-0.09, 0.08) |
| Emotional (Adjusting for instrumental) | Cross-sectional | Partially adjusted | 0.06 (-0.10, 0.23) |
| Instrumental (Adjusting for emotional) | Cross-sectional | Fully adjusted | -0.05 (-0.20, 0.10) |
| Emotional (Adjusting for instrumental) | Cross-sectional | Fully adjusted | 0.02 (-0.08, 0.13) |
| Instrumental (Adjusting for emotional) | Longitudinal | Partially adjusted | -0.00 (-0.08, 0.07) |
| Emotional (Adjusting for instrumental) | Longitudinal | Partially adjusted | 0.06 (-0.06, 0.18) |
| Instrumental (Adjusting for emotional) | Longitudinal | Fully adjusted | -0.05 (-0.23, 0.12) |
| Emotional (Adjusting for instrumental) | Longitudinal | Fully adjusted | 0.06 (-0.03, 0.15) |

**
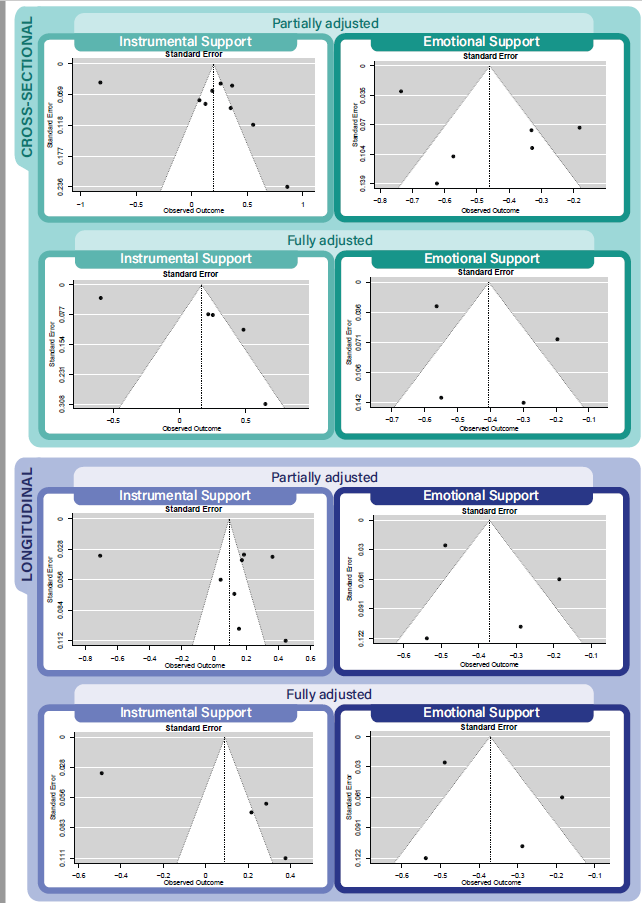
**

# **Figure S1.** Funnel plot for the meta-analysis examining association between emotional and instrumental social support (separate models) and depression scores a) cross-sectionally and b) longitudinally.

**
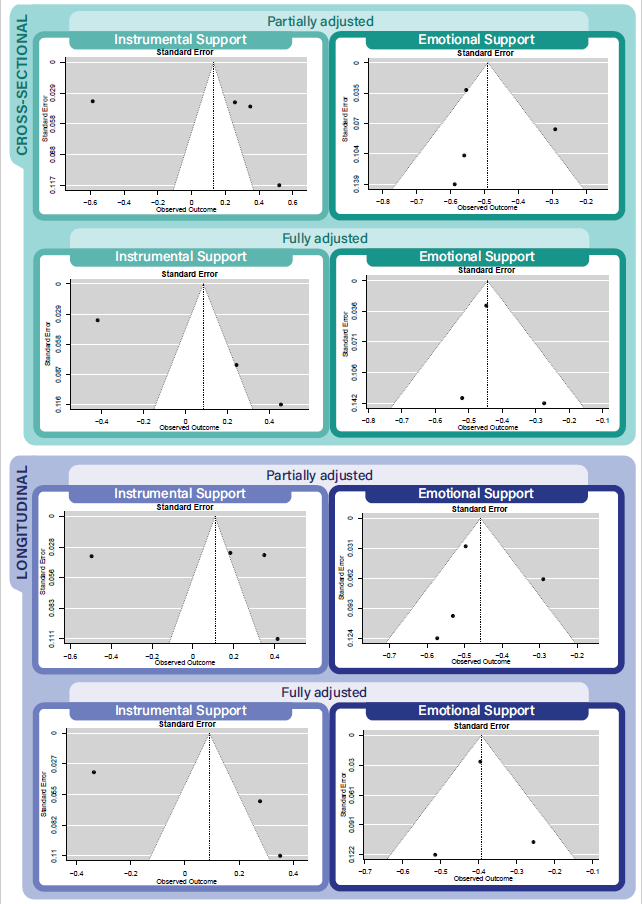
**

# Figure S2. Funnel plot for the meta-analysis examining association between emotional and instrumental social support (combined model) and depression scores a) cross-sectionally and b) longitudinally.

# References for Supplementary Material

1. Castro-Costa É, Fuzikawa C, Uchoa E, Firmo JOA, Lima-Costa MF. Norms for the mini-mental state examination: adjustment of the cut-off point in population-based studies (evidences from the Bambuí health aging study). *Arquivos de neuro-psiquiatria*. 2008;66(3A):524-528.

2. Sullivan KJ, Liu A, Chang CCH, et al. Alzheimer’s disease pathology in a community-based sample of older adults without dementia: The MYHAT neuroimaging study. *Brain Imaging and Behavior*. Published online 2020:1-9.

3. Krishna M, Kumar GM, Veena SR, et al. Birth size, risk factors across life and cognition in late life: protocol of prospective longitudinal follow-up of the MYNAH (MYsore studies of Natal effects on Ageing and Health) cohort. *BMJ open*. 2017;7(2):e012552.
